# 1,3-Bis(1*H*-benzimidazol-2-yl)-2-oxapropane

**DOI:** 10.1107/S1600536809011507

**Published:** 2009-04-02

**Authors:** Ying Chen, Jixi Guo, Ruirui Yun, Huilu Wu

**Affiliations:** a School of Chemical and Biological Engineering, Lanzhou Jiaotong University, Lanzhou 730070, People’s Republic of China; b Institute of Applied Chemistry, Xinjiang University, Urumqi 830046, Xinjiang, People’s Republic of China

## Abstract

The title molecule, C_16_H_14_N_4_O, lies on a crystallographic inversion center. The –CH_2_– groups and the O atom are disordered over two sites with equal occupancy, the disorder of the O atom being symmetry imposed. In the crystal structure, mol­ecules are linked into a two-dimensional network parallel to (001) *via* inter­molecular N—H⋯N hydrogen bonds.

## Related literature

For the applications of bis­(2-benzimidazol­yl)alkanes, see: Cai *et al.* (2003 ▶); Min & Suh (2000 ▶); Roderick *et al.* (1972 ▶). For the isostructural amine analog, see: Tarazon Navarro & McKee (2003 ▶).
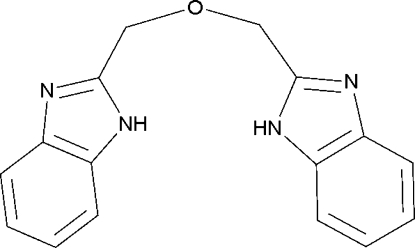


## Experimental

### 

#### Crystal data

C_16_H_14_N_4_O
*M* *_r_* = 278.31
Orthorhombic, 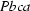

*a* = 8.2143 (4) Å
*b* = 9.6296 (3) Å
*c* = 16.8088 (7) Å
*V* = 1329.58 (9) Å^3^
*Z* = 4
Mo *K*α radiation
μ = 0.09 mm^−1^
*T* = 153 K
0.19 × 0.13 × 0.09 mm

#### Data collection

Rigaku R-AXIS Spider diffractometer
Absorption correction: multi-scan (*ABSCOR*; Higashi, 1995 ▶) *T*_min_ = 0.983, *T*_max_ = 0.992
11836 measured reflections
1525 independent reflections
1189 reflections with *I* > 2σ(*I*)
*R*_int_ = 0.045

#### Refinement

*R*[*F* ^2^ > 2σ(*F* ^2^)] = 0.043
*wR*(*F* ^2^) = 0.112
*S* = 1.08
1525 reflections
108 parameters
H atoms treated by a mixture of independent and constrained refinement
Δρ_max_ = 0.22 e Å^−3^
Δρ_min_ = −0.20 e Å^−3^

### 

Data collection: *RAPID-AUTO* (Rigaku/MSC, 2004 ▶); cell refinement: *RAPID-AUTO*; data reduction: *RAPID-AUTO*; program(s) used to solve structure: *SHELXS97* (Sheldrick, 2008 ▶); program(s) used to refine structure: *SHELXL97* (Sheldrick, 2008 ▶); molecular graphics: *SHELXTL* (Sheldrick, 2008 ▶); software used to prepare material for publication: *SHELXTL*.

## Supplementary Material

Crystal structure: contains datablocks global, I. DOI: 10.1107/S1600536809011507/lh2795sup1.cif

Structure factors: contains datablocks I. DOI: 10.1107/S1600536809011507/lh2795Isup2.hkl

Additional supplementary materials:  crystallographic information; 3D view; checkCIF report

## Figures and Tables

**Table 1:** Hydrogen-bond geometry (Å, °)

*D*—H⋯*A*	*D*—H	H⋯*A*	*D*⋯*A*	*D*—H⋯*A*
N2—H2*N*⋯N1^i^	0.953 (19)	1.951 (19)	2.8803 (16)	164.2 (15)

Symmetry code: (i) 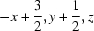.

**Fig. 1.**
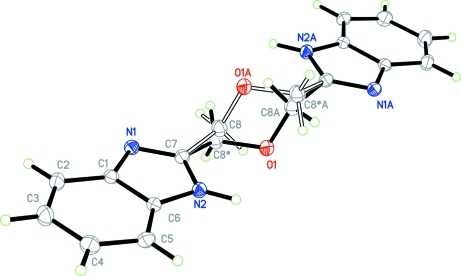
The molecular structure of the title compound with displacement ellipsoids shown at the 30% probability level. Open bonds indicate the disorder component. Symmetry code (A): -x+2, -y+1, -z+1.

**Fig. 2.**
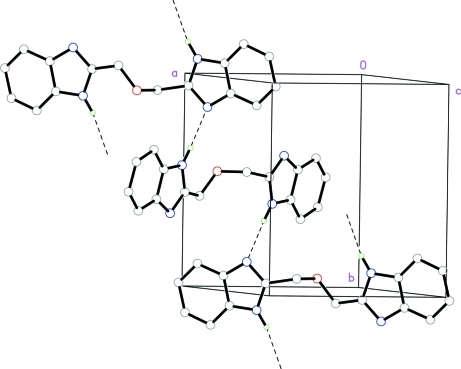
Part of the crystal structure of the title compound with hydrogen bonds shown as dashed lines. Only H atoms involed in hydrogen bonds are shown. The disorder is not shown.

## supplementary crystallographic information

### Comment

Interest in bis(2-benzimidazolyl)alkanes and their derivatives are widespread and has originated from their wide-ranging anti-viral activity and their importance in selective ion-exchange resins (Cai *et al.*,  2003; Min *et al.*, 2000; Roderick *et al.*,  1972). The molecular structure of the title compound is shown in Fig. 1 and is isostructural with the amine analog (Tarazon Navarro & McKee, 2003). In the crystal structure, molecules are linked into a two-dimensional network parallel to the (001) plane via intermolecular N-H···N hydrogen bonds (Fig. 2).

### Experimental

21.44 g (160 mmol) of diglycolic acid was combined with 34.56 g (320 mmol) of *o*-phenylenediamine in 350 ml of 5 N HCl. The solution was refluxed for 24 h. The resulting solution was neutralized with NH_4_OH. The white precipitate was collected, washed with MeOH and absolute Et_2_O, and dried *in vacuo*. The dried precipitate was dissolved in DMF to a green solution and through the ether diffusion exhalation crystal after three days at room temperature. The colorless crystals suitable for X-ray diffraction studies were obtained after four weeks. Yield, 29.36 g (66%). (found: C, 68.78; H, 5.09; N,20.51 Calcd. for C16H14N4O: C, 69.05; H, 5.07; N, 20.13)

### Refinement

H atoms were included in calclulated positions and refined in a riding-model approximation with C—H distances ranging from 0.95 to 0.96Å and *U*_iso_(H) = 1.2 *U*_eq_(C). The H atom bonded to N2 was refined independently with an isotropic displacement parameter. The -CH_2_ groups and the O atom are disordered over two sites with equall occupancy, the disorder of the O atom being symmetry imposed. The anisotropic displacement parameters of C8 and C8* were constrained to be equall using the EADP instruction in SHELXL (Sheldrick, 2008).

### Crystal data

**Table:**

C_16_H_14_N_4_O	*F*(000) = 584
*M**_r_* = 278.31	*D*_x_ = 1.390 Mg m^−^^3^
Orthorhombic, *P**b**c**a*	Mo *K*α radiation, λ = 0.71073 Å
Hall symbol: -P 2ac 2ab	Cell parameters from 1231 reflections
*a* = 8.2143 (4) Å	θ = 3.5–25.5°
*b* = 9.6296 (3) Å	µ = 0.09 mm^−^^1^
*c* = 16.8088 (7) Å	*T* = 153 K
*V* = 1329.58 (9) Å^3^	Prism, colourless
*Z* = 4	0.19 × 0.13 × 0.09 mm

### Data collection

**Table:**

Rigaku R-AXIS Spider diffractometer	1525 independent reflections
Radiation source: fine-focus sealed tube	1189 reflections with *I* > 2σ(*I*)
graphite	*R*_int_ = 0.045
φ and ω scans	θ_max_ = 27.5°, θ_min_ = 3.5°
Absorption correction: multi-scan (*ABSCOR*; Higashi, 1995)	*h* = −10→10
*T*_min_ = 0.983, *T*_max_ = 0.992	*k* = −12→11
11836 measured reflections	*l* = −20→21

### Refinement

**Table:**

Refinement on *F*^2^	Secondary atom site location: difference Fourier map
Least-squares matrix: full	Hydrogen site location: inferred from neighbouring sites
*R*[*F*^2^ > 2σ(*F*^2^)] = 0.043	H atoms treated by a mixture of independent and constrained refinement
*wR*(*F*^2^) = 0.112	*w* = 1/[σ^2^(*F*_o_^2^) + (0.0566*P*)^2^ + 0.3224*P*] where *P* = (*F*_o_^2^ + 2*F*_c_^2^)/3
*S* = 1.08	(Δ/σ)_max_ < 0.001
1525 reflections	Δρ_max_ = 0.22 e Å^−^^3^
108 parameters	Δρ_min_ = −0.20 e Å^−^^3^
0 restraints	Extinction correction: *SHELXL97* (Sheldrick, 2008), Fc^*^=kFc[1+0.001xFc^2^λ^3^/sin(2θ)]^-1/4^
Primary atom site location: structure-invariant direct methods	Extinction coefficient: 0.015 (3)

### Special details

**Table:**

Geometry. All esds (except the esd in the dihedral angle between two l.s. planes) are estimated using the full covariance matrix. The cell esds are taken into account individually in the estimation of esds in distances, angles and torsion angles; correlations between esds in cell parameters are only used when they are defined by crystal symmetry. An approximate (isotropic) treatment of cell esds is used for estimating esds involving l.s. planes.
Refinement. Refinement of F^2^ against ALL reflections. The weighted R-factor wR and goodness of fit S are based on F^2^, conventional R-factors R are based on F, with F set to zero for negative F^2^. The threshold expression of F^2^ > 2sigma(F^2^) is used only for calculating R-factors(gt) etc. and is not relevant to the choice of reflections for refinement. R-factors based on F^2^ are statistically about twice as large as those based on F, and R- factors based on ALL data will be even larger.

### Fractional atomic coordinates and isotropic or equivalent isotropic displacement parameters (Å^2^)

**Table:**

	*x*	*y*	*z*	*U*_iso_*/*U*_eq_	Occ. (<1)
O1	0.9632 (3)	0.5599 (2)	0.53873 (12)	0.0332 (5)	0.50
N1	0.64853 (14)	0.36549 (11)	0.43694 (6)	0.0256 (3)	
N2	0.71007 (14)	0.59185 (11)	0.43361 (7)	0.0254 (3)	
H2N	0.770 (2)	0.675 (2)	0.4419 (10)	0.049 (5)*	
C1	0.54372 (16)	0.43254 (13)	0.38456 (8)	0.0245 (3)	
C2	0.41904 (18)	0.37997 (14)	0.33731 (9)	0.0329 (4)	
H2A	0.3907	0.2844	0.3391	0.039*	
C3	0.3378 (2)	0.47100 (16)	0.28783 (10)	0.0391 (4)	
H3A	0.2525	0.4372	0.2549	0.047*	
C4	0.3787 (2)	0.61227 (16)	0.28509 (9)	0.0366 (4)	
H4A	0.3217	0.6717	0.2496	0.044*	
C5	0.49977 (17)	0.66712 (14)	0.33267 (8)	0.0303 (3)	
H5A	0.5257	0.7633	0.3319	0.036*	
C6	0.58166 (16)	0.57462 (13)	0.38169 (7)	0.0237 (3)	
C7	0.74397 (17)	0.46457 (12)	0.46425 (8)	0.0254 (3)	
C8	0.8957 (12)	0.4403 (12)	0.5122 (5)	0.0319 (12)	0.50
H8A	0.8872	0.3484	0.5339	0.038*	0.50
H8B	0.9052	0.5059	0.5549	0.038*	0.50
C8*	0.8603 (12)	0.4441 (12)	0.5307 (5)	0.0319 (12)	0.50
H8*A	0.8049	0.4226	0.5795	0.038*	0.50
H8*B	0.9273	0.3657	0.5177	0.038*	0.50

### Atomic displacement parameters (Å^2^)

**Table:**

	*U*^11^	*U*^22^	*U*^33^	*U*^12^	*U*^13^	*U*^23^
O1	0.0271 (11)	0.0310 (10)	0.0415 (11)	−0.0002 (8)	−0.0057 (9)	−0.0077 (8)
N1	0.0253 (6)	0.0221 (6)	0.0293 (6)	0.0006 (4)	0.0002 (5)	0.0014 (4)
N2	0.0249 (6)	0.0218 (6)	0.0297 (6)	−0.0011 (5)	−0.0015 (5)	0.0011 (4)
C1	0.0220 (7)	0.0231 (7)	0.0283 (7)	0.0009 (5)	0.0033 (5)	0.0007 (5)
C2	0.0314 (8)	0.0266 (7)	0.0406 (8)	−0.0031 (6)	−0.0052 (6)	−0.0021 (6)
C3	0.0342 (9)	0.0363 (8)	0.0469 (9)	−0.0002 (6)	−0.0148 (7)	−0.0009 (6)
C4	0.0346 (8)	0.0344 (8)	0.0407 (8)	0.0067 (6)	−0.0091 (7)	0.0039 (6)
C5	0.0312 (8)	0.0225 (7)	0.0372 (7)	0.0037 (5)	−0.0002 (6)	0.0016 (5)
C6	0.0208 (7)	0.0230 (6)	0.0273 (6)	0.0008 (5)	0.0010 (5)	−0.0009 (5)
C7	0.0262 (7)	0.0221 (7)	0.0279 (6)	0.0010 (5)	−0.0001 (5)	0.0012 (5)
C8	0.029 (4)	0.0320 (9)	0.034 (4)	−0.001 (2)	−0.004 (2)	0.002 (2)
C8*	0.029 (4)	0.0320 (9)	0.034 (4)	−0.001 (2)	−0.004 (2)	0.002 (2)

### Geometric parameters (Å, °)

**Table:**

O1—C8*	1.405 (12)	C4—C5	1.381 (2)
O1—C8^i^	1.441 (7)	C4—H4A	0.9500
N1—C7	1.3175 (17)	C5—C6	1.3874 (18)
N1—C1	1.3904 (17)	C5—H5A	0.9500
N2—C7	1.3583 (16)	C7—C8*	1.483 (12)
N2—C6	1.3790 (17)	C7—C8	1.502 (12)
N2—H2N	0.953 (19)	C8—O1^i^	1.441 (7)
C1—C2	1.3914 (19)	C8—H8A	0.9600
C1—C6	1.4041 (19)	C8—H8B	0.9600
C2—C3	1.380 (2)	C8*—O1^i^	1.862 (7)
C2—H2A	0.9500	C8*—H8*A	0.9600
C3—C4	1.402 (2)	C8*—H8*B	0.9600
C3—H3A	0.9500		
			
C8—O1—C8^i^	97.6 (5)	N1—C7—C8	124.6 (5)
C8*—O1—C8^i^	115.2 (7)	N2—C7—C8	120.9 (5)
C8—O1—C8*^i^	95.4 (5)	O1—C8—C7	112.6 (8)
C8*—O1—C8*^i^	113.1 (3)	O1^i^—C8—C7	110.5 (6)
C8^i^—O1—H8B	136.0	O1—C8—H8A	133.4
C8*^i^—O1—H8B	134.6	O1^i^—C8—H8A	106.5
C7—N1—C1	104.64 (10)	C7—C8—H8A	106.7
C7—N2—C6	106.76 (11)	O1^i^—C8—H8B	112.3
C7—N2—H2N	126.8 (11)	C7—C8—H8B	111.5
C6—N2—H2N	126.2 (11)	H8A—C8—H8B	109.1
N1—C1—C2	130.41 (12)	O1—C8—H8*A	93.2
N1—C1—C6	109.68 (12)	O1^i^—C8—H8*A	158.6
C2—C1—C6	119.88 (12)	C7—C8—H8*A	90.6
C3—C2—C1	117.95 (13)	H8A—C8—H8*A	62.0
C3—C2—H2A	121.0	H8B—C8—H8*A	60.4
C1—C2—H2A	121.0	O1—C8—H8*B	128.0
C2—C3—C4	121.35 (14)	O1^i^—C8—H8*B	78.4
C2—C3—H3A	119.3	C7—C8—H8*B	119.4
C4—C3—H3A	119.3	H8B—C8—H8*B	119.7
C5—C4—C3	121.63 (14)	O1—C8*—C7	110.8 (7)
C5—C4—H4A	119.2	O1—C8*—H8A	128.6
C3—C4—H4A	119.2	C7—C8*—H8A	108.8
C4—C5—C6	116.58 (13)	H8A—C8*—H8B	125.3
C4—C5—H5A	121.7	O1—C8*—H8*A	111.9
C6—C5—H5A	121.7	C7—C8*—H8*A	111.5
N2—C6—C5	132.03 (12)	O1^i^—C8*—H8*A	154.1
N2—C6—C1	105.38 (11)	O1—C8*—H8*B	107.5
C5—C6—C1	122.58 (13)	C7—C8*—H8*B	107.6
N1—C7—N2	113.54 (12)	H8B—C8*—H8*B	115.5
N1—C7—C8*	123.3 (5)	H8*A—C8*—H8*B	107.3
N2—C7—C8*	122.5 (5)		

Symmetry codes: (i) −*x*+2, −*y*+1, −*z*+1.

### Hydrogen-bond geometry (Å, °)

**Table:**

*D*—H···*A*	*D*—H	H···*A*	*D*···*A*	*D*—H···*A*
N2—H2N···N1^ii^	0.953 (19)	1.951 (19)	2.8803 (16)	164.2 (15)

Symmetry codes: (ii) −*x*+3/2, *y*+1/2, *z*.
